# Synthesis of fullerene nanowhiskers using the liquid–liquid interfacial precipitation method and their mechanical, electrical and superconducting properties

**DOI:** 10.1088/1468-6996/16/1/013502

**Published:** 2015-02-25

**Authors:** Kun’ichi Miyazawa

**Affiliations:** Fullerene Engineering Group, Materials Processing Unit, National Institute for Materials Science, Tsukuba, Ibaraki 305-0044, Japan

**Keywords:** fullerene nanowhisker, fullerene nanotube, fullerene nanosheet, fullerene nanofiber, LLIP method, superconductor

## Abstract

Fullerene nanowhiskers (FNWs) are thin crystalline fibers composed of fullerene molecules, including C_60_, C_70_, endohedral, or functionalized fullerenes. FNWs display *n*-type semiconducting behavior and are used in a diverse range of applications, including field-effect transistors, solar cells, chemical sensors, and photocatalysts. Alkali metal-doped C_60_ (fullerene) nanowhiskers (C_60_NWs) exhibit superconducting behavior. Potassium-doped C_60_NWs have realized the highest superconducting volume fraction of the alkali metal-doped C_60_ crystals and display a high critical current density (*J*_c_) under a high magnetic field of 50 kOe. The growth control of FNWs is important for their success in practical applications. This paper reviews recent FNWs research focusing on their mechanical, electrical and superconducting properties and growth mechanisms in the liquid–liquid interfacial precipitation method.

## 1. Introduction

Fullerene molecules consist of closed cage-type structures that are composed of carbon atoms. The best-known fullerene is C_60_, which was discovered by Kroto *et al* in 1985 [1]. The second well-known molecule is C_70_, which was also identified in [1]. The C_60_ molecule is analogous to a soccer ball with 12 pentagons and 60 vertices where carbon atoms are located, and has 30 six-membered ring/six-membered ring joints with double bonds of carbon and 60 five-membered ring/six-membered ring joints with single bond of carbon.

Polymerization of C_60_ molecules can occur via [2 + 2] cycloaddition reactions, which form four-membered rings between adjacent C_60_ molecules. This cycloaddition mechanism involves a change of carbon hybridization from sp^2^ to sp^3^ [2].

Various properties of C_60_ have been studied by forming thin films on suitable substrates. Bulk samples can also be prepared by sintering at high temperatures. The [2 + 2] cycloaddition polymerization of C_60_ molecules is known to occur in the presence of ultraviolet or visible light illumination [3, 4], high-pressure sintering [5–8], and electron beam irradiation [9, 10]. The hardness of high-pressure sintered C_60_ reaches 200–300 GPa [11, 12].

However, fine needle-like crystals (whiskers) comprising C_60_, ‘C_60_ (fullerene) nanowhiskers (C_60_NWs)’, were found in a colloidal solution of lead zirconate titanate (PZT) with C_60_ added [13–15].

Fullerene nanofibers are linear and thin, with diameters less than 1000 nm [16, 17]. Fullerene nanosheets are thin two-dimensional substances. In this paper, we define fullerene nanosheets to be less than 1000 nm in thickness. Fullerene nanofibers and nanosheets can include a variety of fullerene molecules and their derivatives including C_60_, C_70_, Sc_3_N@C_80_ [18], C_60_[C(COOC_2_H_5_)_2_] [19–21] and (*η*^2^-C_60_)Pt(PPh_3_)_2_ [22].

The aspect ratio of fullerene nanofibers is defined to be greater than three [16]. Fullerene nanofibers are described as either non-tubular or tubular [23–27]. Non-tubular crystalline fullerene nanofibers are called fullerene nanowhiskers (FNWs). FNWs with both single-crystal and polycrystalline structures have been reported [53].

**Figure 1.**
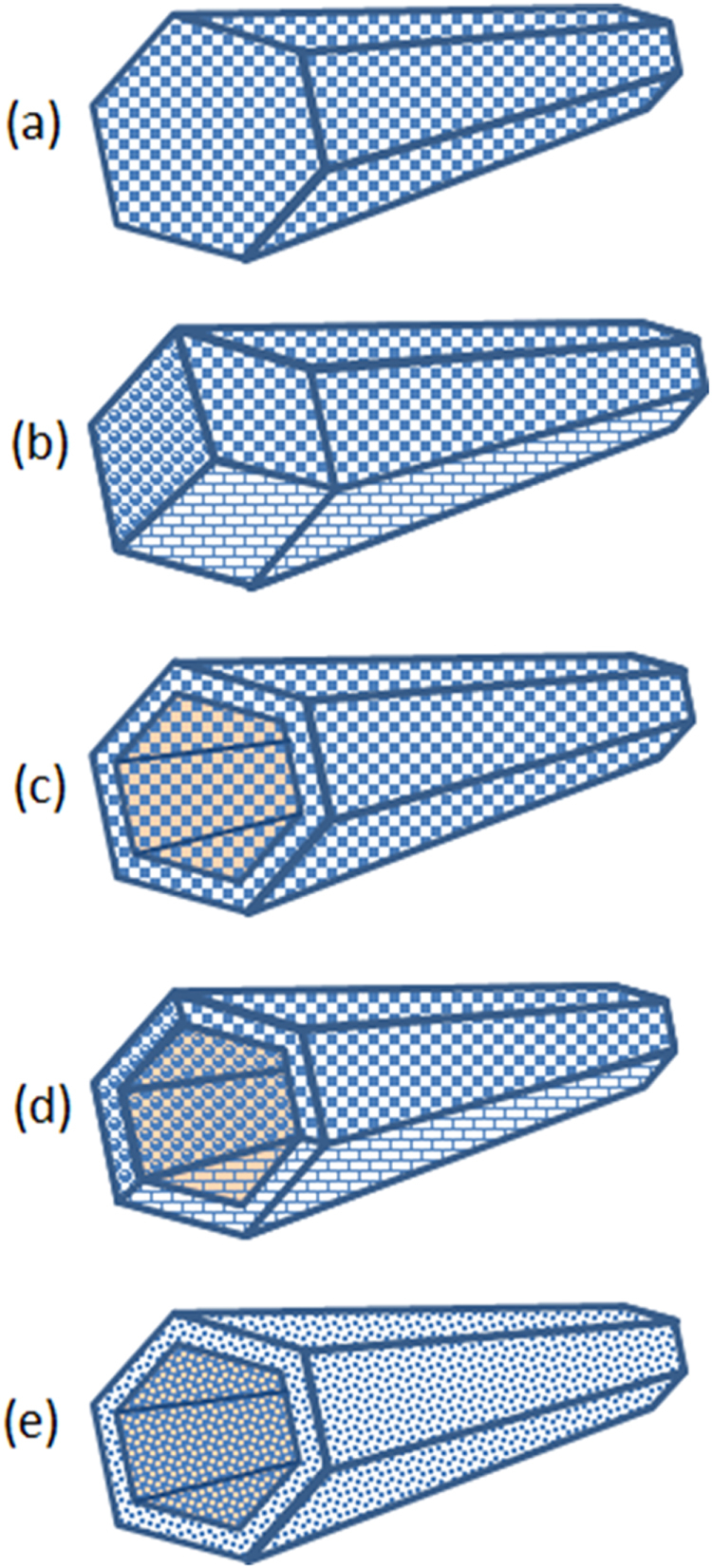
Classification of fullerene nanofibers. (a) single-crystal fullerene nanowhisker, (b) polycrystal fullerene nanowhisker, (c) single-crystal fullerene nanotube, (d) polycrystal fullerene nanotube, (e) amorphous fullerene nanotube.

Fullerene nanofibers can incorporate either one or multiple types of fullerenes. This enables formation of both monocomponent and multicomponent structures. Examples of monocomponent structures include C_60_NWs, C_70_ (fullerene) nanowhiskers (C_70_NWs), C_60_ or C_70_ (fullerene) nanotubes (C_60_NTs or C_70_NTs), [23, 25, 28], and FNWs composed of C_60_[C(COOC_2_H_5_)_2_] molecules (C_60_[C(COOC_2_H_5_)_2_]NWs). Examples of multicomponent fullerene nanofibers include two-component C_60_–C_70_ NWs [29], two-component C_60_–C_70_ NTs [23], two-component C_60_–C_60_[C(COOC_2_H_5_)_2_] NWs [19], and two-component C_60_–(*η*^2^-C_60_)Pt(PPh_3_)_2_ NWs [22]. Figure 1 shows the classification of fullerene nanofibers.

Fullerene nanofibers and nanosheets can be synthesized using the ‘liquid–liquid interfacial precipitation (LLIP) method’ [30], which has been widely applied [31–38]. In this review, we discuss the LLIP method to synthesize fullerene nanofibers and nanosheets and the applications in which these materials have been investigated.

The terminology ‘FNW’ represents all needle-like crystals comprising fullerene molecules with diameters less than 1000 nm. The words ‘nanorod’ and ‘nanowire’ are replaced with ‘nanowhisker’ to avoid confusion as was described in review paper [16].

## 2. Synthesis of FNWs

### 2.1. LLIP method

The LLIP method is commonly used to synthesize fullerene nanofibers and nanosheets [30]. This method relies on diffusion of a poor solvent of fullerenes such as isopropyl alcohol (IPA) into a fullerene-saturated toluene solution. An aliquot of a C_60_-saturated toluene solution is added to a glass bottle. Following this, an appropriate amount of IPA is added gently to the solution to form a liquid–liquid interface [8]. The resulting mixture is kept at ambient temperatures, typically below 25 °C. During the slow mixing of toluene and IPA, the liquid–liquid interface becomes supersaturated in C_60_ and allows nucleation of C_60_NWs to occur. This supersaturated state is maintained as IPA diffuses into toluene and assists in the growth of C_60_NWs. This procedure is named ‘static LLIP method’ [30, 39]. The glass bottle is kept still in an incubator, where the C_60_NWs self-assemble into a shape similar to a cotton ball. The LLIP method can also be used in combination with ultrasonic mixing, manual mixing, or injection [24, 39, 40]. Ultrasonication induces rapid mixing of good solvents and poor solvents, causing formation of fine fullerene nuclei that grow into fullerene nanofibers or nanosheets.

The static LLIP method can involve either layering a poor solvent onto a good solvent or vice versa, and can be combined with manual mixing, supersonic mixing, mixing by injection of liquid, or ultrasonic mixing of liquid droplets [41]. These methods are collectively named the ‘dynamic LLIP method’.

Cha *et al* and Miyazawa *et al* reported the diaphragm LLIP method (DLLIP method), which involves injecting a poor solvent for fullerene into a fullerene solution through a porous membrane [40, 42, 43]. As an example, if IPA is slowly injected into a C_60_-saturated toluene solution through an anodic aluminum oxide membrane with nanosized pores, vertically grown microtubes of C_60_ are produced. All methods that mix two solvents to form fullerene nanofibers and nanosheets can be classified as LLIP processes.

Using the DLLIP method, the influence of alcohol chain length (methanol, ethanol, and IPA) on the length of C_60_ whiskers was investigated using toluene as a good solvent for C_60_. Amer *et al* reported that the length of C_60_ whiskers decreased when the chain length of the alcohol (poor solvent) increased [44]. The temperatures at which the C_60_ whiskers were grown was not reported; however, the above result suggests that the chain length of the alcohol influences the desolvation energy of solvated C_60_ molecules that governs the rate-limiting process of surface reaction [45].

### 2.2. Growth mechanism of FNWs using the LLIP method

The Young modulus of C_60_NWs has been examined using a transmission electron microscope equipped with an atomic force microscope [46]. The Young modulus of C_60_NWs increases with decreasing diameter [46–49]. This phenomenon is thought to occur because C_60_NWs have a core–shell structure with a porous interior region and a dense surface region [48, 50]. Kizuka *et al* found that C_70_NWs containing solvent molecules had a higher density of lattice defects in their interior regions, which caused a reduction in the Young modulus [51]. Additionally, the Young modulus of C_70_NTs was found to increase with decreasing diameter [25]. These studies conclude that by decreasing the diameter of fullerene nanofibers, crystallinity is increased, which in turn leads to an increase in the Young modulus.

In the LLIP process, FNWs grow from seed crystals [52–54]. The size of the initial C_60_NW nuclei is influenced by the degree of supersaturation of C_60_ in solution, which is determined by the mixing ratio of both good and poor solvents [39]. C_60_NTs grow in both directions along their growth axis from the seed crystals [53, 54]. However, the seed crystals should disappear by the core dissolution mechanism to form a through-hole structure [55].

The re-growth of C_60_NTs was observed in ultrasonically pulverized C_60_NTs [53]. The ultrasonically fractured C_60_NTs have steep wall edges, on which C_60_ molecules accumulate and crystallize [53]. This preferential accumulation of C_60_ in areas with a small radius of curvature, such as the hexagonal vertices, is an important growth mechanism of fullerene nanotubes [53, 54].

The growth of C_60_NWs is influenced by numerous factors, including time, temperature, light, solvent species, the ratio between good and poor solvents, and contained impurity water [39, 56–59]. The growth mechanism of C_60_NWs in C_60_-saturated toluene and IPA has been studied closely. The activation energy of growth (52.8 kJ mol^−1^) was calculated by varying the temperature and measuring the length of C_60_NWs. This value is approximately four times greater than the value obtained for the diffusion of C_60_ in a mixed solution of toluene and acetonitrile (13.1 kJ mol^−1^, 4:1 v/v) [56, 60]. The high activation energy indicated that the growth of C_60_NWs is rate limited by the desolvation process of C_60_ molecules bonded with solvent molecules on the crystal surface.

The dynamic LLIP process involves a fullerene solution being forcibly mixed with a poor solvent for fullerene. This process generates microscopic liquid–liquid interfaces between the fullerene solution and the poor solvent of fullerene, where supersaturated solutions lead to rapid nucleation of fine fullerene crystals. The formation of granular, linear, or sheet fullerene crystal morphologies depends on the growth kinetics, which may be governed by the degree of supersaturation, solvent species, and temperature.

**Figure 2.**
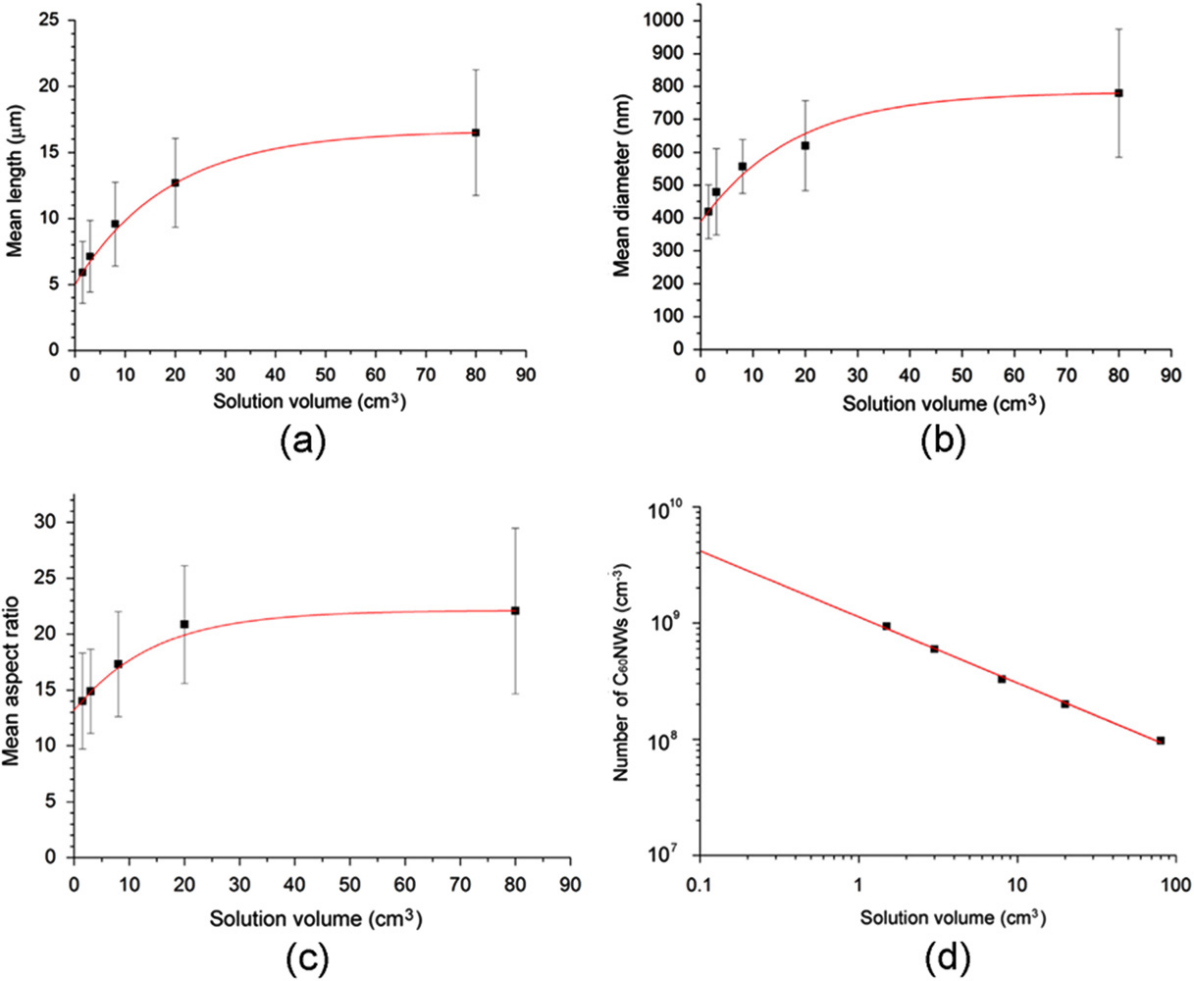
(a) Relationship between solution volume and mean length of C_60_NWs. The equation fitted to the data is *y* = −11.6exp(−*x*/18.7) + 16.6. (b) Relationship between solution volume and mean diameter of C_60_NWs. The equation fitted to the data is *y* = −396.6exp(−*x*/17.6) + 783.7. (c) Relationship between solution volume and the mean aspect ratio of C_60_NWs. The equation fitted to the data is *y* = −9exp(−*x*/14.2) + 22.1. (d) Estimated number of C_60_NWs per unit volume plotted versus the solution volume. The equation fitted to the data is *y* = −1.12496 × 10^9^ *x*^−0.5674^. Reprinted from [62], copyright 2014, with permission from Elsevier.

Size control of fullerene nanofibers is critical for practical applications. Wakahara *et al* reported that the diameter of C_60_NWs varied with the size of the glass bottles used in their synthesis. Linear relationships between the area of the liquid–liquid interface and the diameter of C_60_NWs were observed when the total volume of solution was fixed [61]. Changes in the lengths and diameters of C_60_NWs upon varying the solution volume have been examined [62]. These C_60_NWs were prepared by dynamic LLIP in a C_60_-saturated toluene and IPA system. After the initial formation of a liquid–liquid interface by layering an equal amount of IPA on a C_60_-saturated toluene solution, the solution was manually mixed by shaking 30 times. The relationships between solution volume and mean length, diameter and aspect ratio are shown in figures 2(a)–(c) [62]. The aspect ratio, as derived from the *y*-intercepts of figures 2(a) and (b) (5.02 *μ*m/387 nm) yielded a value of 13.0, almost identical to the value derived from the *y*-intercept of figure 2(c) (13.1). Hence, it is reasonable to consider the size of C_60_NW nuclei can be estimated using the relationships shown in figures 2(a)–(c).

The relationship between the solution volume and number of C_60_NWs per unit volume is shown in figure 2(d). The number density, as calculated from the nominal content of C_60_ and the mean size of C_60_NWs in solution [62], increased as the solution volume decreased. This implies that the volume fraction of liquid–liquid interfaces increases when the solution volume is decreased. A power law relationship (y = 1.12 × 10^9^*x*^−0.567^) was fitted to the data with an approximate index of −0.5, showing that the number density of C_60_NW nuclei in solution is inversely proportional to the square root of the solution volume.

**Figure 3.**
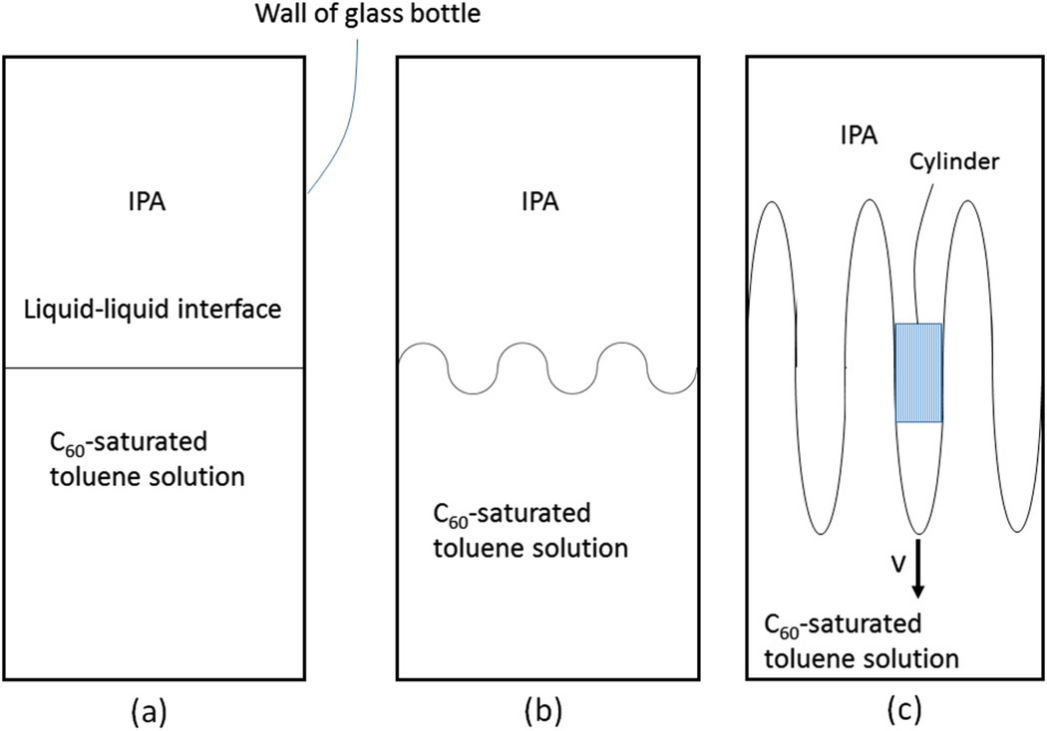
Model showing the liquid–liquid interface (a) changing with manual mixing (b). The interface front between the C_60_-saturated toluene solution and IPA is assumed to move with a velocity v along the vertical direction of the glass bottle (c).

**Figure 4.**
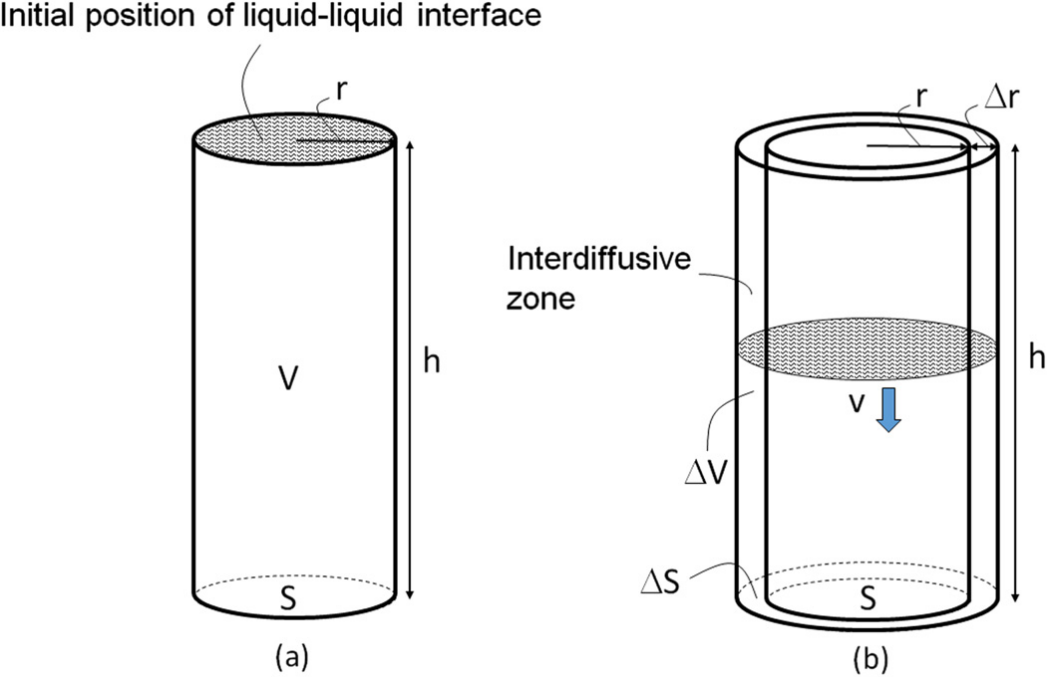
Cylindrical model used to calculate the number density N of C_60_NW nuclei for the region of the liquid–liquid interface shown in figure 3(c).

A model describing the changes in the liquid–liquid interface upon manual mixing is shown in figure 3. The initial layered interface (figure 3(a)) is assumed to form a sinusoidally modulated interface (figure 3(b)) upon the manual mixing. The amplitude of this interface increases along the height of the glass bottle, a section of this wavefront is highlighted by the blue rectangle (figure 3(c)). This highlighted section is modeled by a cylinder with height *h*, radius *r*, basal area *S*, and volume *V* (figure 4(a)). The front of the liquid–liquid interface travels vertically with a velocity *v*.

The following equations hold.1
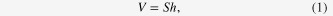

2
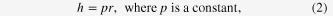

3
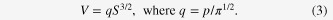
The front of liquid–liquid interface is assumed to move along the height *h* with a time *t*.4
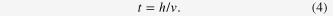
As the liquid–liquid interface front moves, interdiffusion between C_60_-satulated toluene solution and IPA occurs (figure 4(b)). If the values of both *t* and *Δr* are assumed to be small, the area of the interdiffusion zone (*ΔS*) is approximated as follows:5
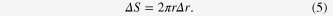
The volume of interdiffusion *ΔV* is:6
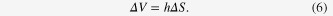
If *Δr* is assumed to be proportional to (*Dt*)^1/2^ with a coefficient of interdiffusion *D* [63], it is calculated as in equation (7) with a constant *a*,7
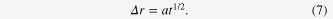



Hence, combining (4), (5), and (7),9
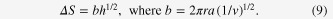
If *N* is defined as the number of C_60_NW nuclei per unit volume in the zone of interdiffusion, the mean number of C_60_NW nuclei contained in a unit volume of a cylinder (*ρ*) can be calculated by combining equations (1), (3), (6), and (9):12
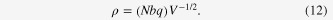



This model suggests that the mean number density of C_60_NW nuclei is inversely proportional to the square root of the solution volume, which was indeed confirmed experimentally (figure 2(d)).

## 3. Electrical and superconducting properties of C_60_NWs

C_60_NWs display *n*-type semiconducting behavior and are used in a diverse range of applications, including field effect transistors (FETs) [64], solar cells [65, 66], photocatalysts [67], chemical sensors [27], and photosensors [68]. However, Wakahara *et al* recently synthesized ambipolar FETs with C_60_/cobalt–porphyrin hybrid nanosheets using a LLIP method [92].

The carrier mobility of C_60_NWs in a FET was determined to be 2 × 10^−2^ cm^2^ V^−1^ s^−1^ under vacuum [64]. However, the as-synthesized solution-grown C_60_ needle-like crystals exhibited a very high mobility up to 11 cm^2^ V^−1^ s^−1^ [69]. As the measured carrier mobility of C_60_NWs, or needle-like crystals of C_60_, depends largely on the measurement conditions (solvent impurities, oxygen impurity, crystal structure, and lattice defects), electrical properties of the materials were investigated under controlled conditions. Only C_60_NWs with clearly defined chemical and structural properties were used.

**Figure 5.**
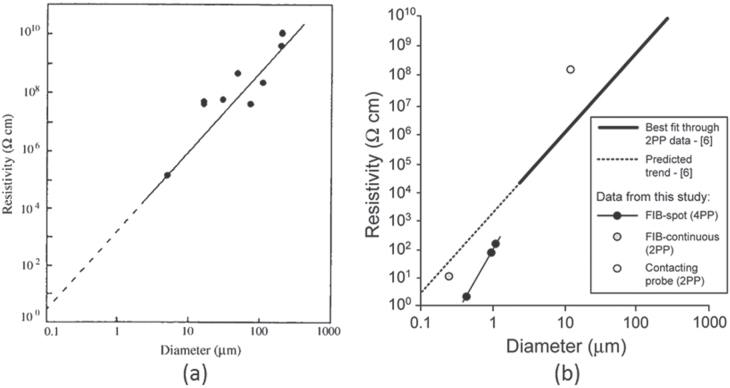
Electrical resistivity of C_60_ whiskers measured as a function of diameter. The resistivity measurement was performed by the two-point probe method (2PP) in (a) and by the four-point probe method (4PP) in (b). [6] in the inset of (b) is identical with [70]. FIB stands for focused ion beam. Part (a) reprinted with permission from [70], copyright © 2003 John Wiley & Sons, Ltd. Part (b) reproduced by permission of ECS—The Electrochemical Society from [71]

The electrical resistivity of C_60_ whiskers with diameters greater than 1 *μ*m (∼10–a few hundred micrometers) was measured using a two-terminal method at ambient temperature [70]. The electrical resistivity of the C_60_ whiskers decreased dramatically with decreasing diameter (figure 5(a)). The resistivity of C_60_NWs is expected to be several Ohm centimeters (*Ω* cm), based on extrapolation of the curve-fitted data. Subsequently, Larsson *et al* measured the electrical resistivity using a four-point probe method [71]. Figure 5(b) summarizes their results including figure 5(a) [70]. The four-point probe method also showed a decrease in resistivity of C_60_ whiskers with decreasing diameter (FIB-spot (4PP)), figure 5(b)). A C_60_NW with a diameter of 650 nm showed a low resistivity of 3 *Ω* cm [71]. The decrease in resistivity with decreasing diameter suggested that C_60_NWs with smaller diameters and shorter C_60_ intermolecular distances are more crystalline and thus have a greater overlap of *π* electrons [70]. Recently, this fact was further confirmed by Barzegar *et al* using thinner C_60_NWs [93]. It was shown that the electrical mobility of as-grown C_60_NWs with diameters less than 300 nm increases with decreasing the diameter of C_60_NWs [64, 93–95].

If the line measured using the four-point probe method is extrapolated to a diameter of 100 nm in figure 5(b), the resistivity will decrease to the order of 10^−3^ *Ω* cm. This result suggests that C_60_NWs may exhibit metallic conductivity when their diameters are sufficiently small. Xu *et al* showed that C_60_NWs are conductive only if the surface is not covered by oxygen [72].

To determine the electrical properties of a semiconductor, it is necessary to measure the temperature dependence of electrical conductivity. Ji *et al* performed these measurements using C_60_NWs with either a face-centered cubic (fcc) or a hexagonal closed packed (hcp) structure [73]. The fcc C_60_NW displayed higher electrical conductivity than did the hcp C_60_NW. This result confirms that the crystal structure influences the electrical properties of C_60_NWs. However, the effect of solvent molecules contained in the hcp C_60_NW is still under some debate. If C_60_ molecules adopt a closely packed structure, a greater overlap between *π* electrons would lead to higher electrical conductivity in these C_60_NWs [70, 73].

Carbon superconductors have been investigated for many years. The superconductivity of graphite (C_8_) specimens doped with alkali metals, including K (superconducting transition temperature (*T*_c_) < 0.55 K [74], 0.128–0.198 K [75]), Cs (*T*_c_ = 0.020–0.135 K [74]), and Rb (*T*_c_ = 0.023–0.151 K [74]) has been reported. Graphite superconductors such as C_6_Ca (*T*_c_ = 11.5 K) and C_6_Yb (*T*_c_ = 6.5 K) were also synthesized [76]. C_60_NWs can be transformed into glassy carbon nanofibers by heat treatment [77–79]. When heated to 3000 °C, C_60_NWs transform into carbon nanofibers with up to 17 graphene layers [77]. The number of stacked graphene layers increases with increasing temperature between 2000 and 3000 °C [77]. Those C_60_NWs heated at high temperatures with developed graphitic ribbons are promising materials that may exhibit superconductivity if doped with alkali metals and alkaline-earth metals. In addition, the high-temperature-treated C_60_NWs become electron emission tips showing striped patterns that reflect the atomic structure of the crumpled graphitic layers [78, 79]. However, since long amorphous carbon nanofibers prepared by high-temperature heat treatment of C_60_NWs showed cytotoxicity like long multiwall carbon nanotubes [96], special care will be necessary in the practical uses of the glassy carbon nanofibers.

In 2004, boron-doped diamond was observed to exhibit superconductivity (*T*_c_ ≈ 4 K) [80]. Takano *et al* found that the *T*_c_ of a boron-doped diamond film was 7.4 K [81].

Hebard *et al* discovered that C_60_ doped with potassium (K) exhibited superconductivity [82]. A superconducting transition temperature (*T*_c_) of 18 K was observed for both K-doped C_60_ films and bulk samples. Tanigaki *et al* reported the highest *T*_c_ value of 33 K in Cs_2_Rb_1_C_60_ powder [83].

**Figure 6.**
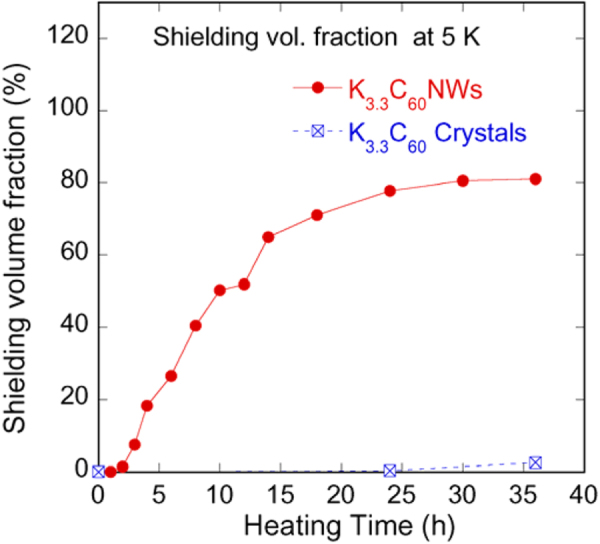
Shielding volume fractions in K-doped C_60_NWs and K-doped C_60_ crystal powder (reprinted from [87]).

Of the three known phases of K-doped C_60_ (fcc (K_3_C_60_), body-centered tetragonal (bct) (K_4_C_60_), and body-centered cubic (bcc) (K_6_C_60_)), only the fcc phase exhibits superconductivity [84]. Although C_60_NWs that are grown in solution display a solvated hexagonal structure, they transform into an fcc structure upon drying and removal of the internal solvent molecules [85]. Hence, these fcc C_60_NWs should be superconducting if doped with alkali metals [15]. C_60_ nanotubes were doped with Li, Na, and K, and the crystal structures were examined using Raman spectroscopy [86]. Superconductive C_60_NWs were also successfully fabricated by doping with K [87, 88]. Although the *T*_c_ value (17 K) of the K-doped C_60_NWs with a nominal composition of K_3.3_C_60_ was lower than the reported value of 18 K [82], the superconducting, shielding volume fraction was as high as 80%, and the critical current density *J*_c_ was more than 3 × 10^5^ A cm^−2^ under 50 kOe [87, 88], although the doping was performed at 200 °C for 24 h. The shielding volume fraction of the K-doped C_60_ crystal powder was less than 1% when doped using the same process (figure 6). The high shielding volume fraction in the K-doped C_60_NWs may allow for light, flexible, and recyclable superconducting carbon cables. Initially, the superconducting shielding volume fraction of K-doped C_60_ crystals was at most 35%, even after prolonged heat treatment (20 days) at temperatures up to 250 °C [89].

Efforts to increase the *T*_c_ value of alkali-doped C_60_NWs are continuing. Values up to 26 K have been achieved by doping with Rb [90]. The volume fraction of Rb-doped C_60_NWs was approximately five times greater than that of Rb-doped C_60_ powder. As Rb is an abundant alkali metal like the other common metals such as copper, lead, or zinc [91], lightweight Rb-doped C_60_NWs are expected to find use in a variety of superconducting applications, including motor cars, cables for power delivery, and wind generators.

## 4. Summary

A variety of fullerene nanofibers and nanosheets have been synthesized using LLIP methods. These materials have found use in a wide range of applications, including solar cells, chemical sensors, photo sensors, photocatalysts, and ambipolar field-effect transistors. The synthesis of C_60_NWs using a dynamic LLIP method with a C_60_-saturated toluene solution and IPA suggests that nucleation is governed by the volume of the liquid–liquid interface produced by interdiffusion between the two solvents.

Alkali-metal-doped C_60_NWs are the first carbon fibers to display superconductivity while being lightweight and flexible. K- or Rb-doped C_60_NWs are promising superconductors with *T*_c_ values that are higher than those of any other practically used metal superconductors. Additionally, they are composed of non-toxic, abundant, and recyclable elements. Fullerene nanomaterials show great promise for a variety of applications in electrical and optical fields.
